# The Main Facts of Dental Jurisprudence

**Published:** 1909-01-15

**Authors:** Charles S. Ayres

**Affiliations:** Oakland, Cal.


### THE MAIN FACTS OF DENTAL JURISPRUDENCE.

BY CHARLES S. AYRES, D.D,S., OAKLAND, CAL.

Jurisprudence is that science which treats of the application of the law, and dental jurisprudence is that branch of jurisprudence which treats of the application of the law to the conditions developed by the practice of dentistry. Thus it is limited on one side by the requirements of the law and on the other by the whole range of dental knowledge. To help a court of law to arrive at a just conclusion in a dental case submitted for decision, anatomy, physiology, therapeutics, materia medica, chemistry, operative and prosthetic dentistry, or any other branch of modern dental education may play an important part, particularly in helping to interpret properly the evidence brought forth.

The jurist, when appealed to for an opinion as to just what protection is afforded the bearer of the title of D. D. S. and a certificate from the State Board of Dental Examiners, will respond that he has the legal right to perform any and all operations within the limitation or domain of dentistry. But just how far the domain of dentistry enters the realm of medicine or oral surgery he will be unable to tell without careful study, for every day the dentist is stepping over some barrier which the day before was considered the boundary line between dentistry and medicine or between dental surgery and oral surgery. Without question the dentist may care for the teeth when sound, treat them when unsound or unhealthy, correct their deformities or malpositions and adapt substitutes for those lost by old age, disease or accident. He may extract or fill teeth, perform other operations upon their alveolar process, and upon the neighboring bones. He is allowed to implant, transplant, or replant teeth and treat diseases of the gums and antrum, either surgically or medically. In short, he must be competent to perform any operation, treat any disease or pathological condition pertaining to the dental organs, and adopt and practice such systematic treatments as are promulgated by the profession.

For years past there has been considerable discussion as to whether or not dentists are permitted to use anesthetics. At this time it seems well established that they can. But the courts will hold the individual accountable for a thorough knowledge of the methods of administration, and the precautions to be observed. In fact, the courts will consider that, in order to prove himself possessed of ordinary skill, he must have the ability to determine if the patient is in a fit condition for anesthetics; he must know the best drug to use in a certain case, and he must have an accurate knowledge of the nature and action of the drug used. If, in case of accident under an anesthetic, the dentist can prove the above qualifications and that he used due caution, he will be held blameless.

What then, is dental malpractice? Rhefus defines it as "bad or unskilled practice in dental surgery, whereby an unskillful operation is performed, the health of the patient injured, or his life destroyed by the improper and careless administration of medicines or anesthetics." Three forms of malpractice are recognized by the law—wilful, negligent and ignorant.

When a dentist performs an operation which he knows and expects will injure the health, damage the person or cause the death of the patient, he is guilty of wilful malpractice.

*Negligent Malpractice* covers those cases where the operator grossly neglects to take those steps which the case demands, as when he neglects to sterilize his instruments and infection ensues.

*Ignorant Malpractice* is committed when a dentist harms a patient by treatment or an operation which a well-educated dentist would consider dangerous or not suited to the case.

The matter of charging for broken engagements has often come up in the courts and it has been decided that the dentist has the right to charge for time actually wasted. That is if he makes an appointment and the patient fails to keep it and he does not use that time for other purposes of his profession, he is entitled to the same fee he would have earned by performing the contemplated operation. If, however, he does use that time operating upon some other person, he cannot collect from the first party. These rulings are justified by the fact that a professional man's time is the capital whereby he earns a livelihood. If a certain part of that is set aside and not used, the one causing the waste by his failure to arrive at the appointed time should compensate the dentist for this loss of capital. But, if there is no loss, there should be no penalty. (*Equity.*)

When a person requests the services of a dentist to be performed upon and for the benefit of another person, he cannot be held responsible for the fee unless he explicitly guarantees in writing the payment thereof. This does not, however, include people within what the law terms the "domestic relation," that is, unless the person requesting the services bears the relation of husband and wife, parent and child or guardian and ward to the one receiving them. In the latter case the mere request for services renders him responsible for the payment of the debt.

If a married woman contracts for necessities to be furnished to a member of the family of herself and husband, the presumption of the law is that she is merely acting as his agent and that he is the responsible party. The wife, however, may assume the responsibility by her express promise binding her separate estate for the payment thereof.

The general law of the liability of infants applies to dental bills. As a rule, a minor is not responsible for any agreement he may enter into, because, if he wishes, he may void the contract by declaring he is not of age. In cases where the parents or guardian refuses to supply those things necessary for life and health, a stranger may order such necessaries and the parent or guardian becomes responsible. Since, then, a minor cannot be absolutely bound by any contract he may enter into it behooves the dentist to make all agreements or contracts with the parent or guardian. If a child comes alone to the office for services, it is best to ask him to bring a note from his parent requesting that the work be done.

When dentistry first became recognized as separate from medicine and for many years thereafter, it was considered that dental work—beyond the mere extraction of an aching tooth— was a luxury to be indulged in only by the wealthy and the fastidious. With the development of the science its usefulness broadened to such an extent that the mass of the people began to realize that it was a luxury no more, but a real necessity to have the teeth repaired, and preserved. The next step was the promulgation and demonstration of the importance to the general health of the thorough mastication of the food. Arrived at this stage, it became apparent that it was a necessity to supply artificial substitutes for missing teeth. As remedial agents and surgical manipulations advanced, the education of the laity took another stride forward, until the courts have held that nearly every operation in the whole range of dental science is a necessity. Therefore, a husband is responsible for professional services for his wife and child and the guardian for his ward.

Perhaps the most intricate and exacting part of the practice of law is that pertaining to the estates of the deceased, for it has been the special care of the courts to protect the rights of those who cannot appear in behalf of themselves or their heirs; but so far as we are concerned—that is the collection of money for work done before death—the law is simple and definite. The itemized account should be sworn to before a notary and filled and presented to the executor within a certain specified time. To be certain of being within this time limit, it is a good plan to render your bill as soon as you learn of the death of the debtor.

When presenting a case in court, much importance is attached to the manner in which the books are kept. Well-kept books are seldom attacked and then almost never proved false. On the other hand, carelessly kept ones are sure to be questioned and often proved inaccurate. Proving the inaccuracy of the books is almost the same as proving their dishonesty, for the dentist will then have to prove the justice of his claim by outside testimony. This, as you know, is a difficult task at the best.

Those books combining the day book and ledger and using signs and symbols instead of plain language are looked upon with disfavor by court and jury if they are not thrown out entirely. Each item should be entered in the day book the same day the services are performed. To carry the greatest weight and give the appearance of honesty and accuracy, the date, item and price should be entered separately and charged to the one who is to pay it. Several fillings or treatments should not be lumped and one charge made for all.

My advice is: Learn what you can and what you cannot do legally. Then, if any case of importance arises, hire a lawyer even if you think you understand the situation perfectly.—*Dental Digest.*

